# Exploring the Synergistic Mechanisms of Nanopulsed Plasma Bubbles and Photocatalysts for Trimethoprim Degradation and Mineralization in Water

**DOI:** 10.3390/nano14100815

**Published:** 2024-05-07

**Authors:** Dimitris Tsokanas, Christos A. Aggelopoulos

**Affiliations:** 1 Laboratory of Cold Plasma and Advanced Techniques for Improving Environmental Systems, Institute of Chemical Engineering Sciences, Foundation for Research and Technology Hellas (FORTH/ICE-HT), 26504 Patras, Greece; 2 Chemistry Department, University of Patras, 26504 Patras, Greece

**Keywords:** cold atmospheric plasma, plasma catalysis, antibiotics, water treatment, zinc oxide, plasma bubbles

## Abstract

In this study, the synergetic action of nanopulsed plasma bubbles (PBs) and photocatalysts for the degradation/mineralization of trimethoprim (TMP) in water was investigated. The effects of ZnO or TiO_2_ loading, plasma gas, and initial TMP concentration were evaluated. The physicochemical characterization of plasma-treated water, the quantification of plasma species, and the use of appropriate plasma species scavengers shed light on the plasma-catalytic mechanism. ZnO proved to be a superior catalyst compared to TiO_2_ when combined with plasma bubbles, mainly due to the increased production of ⋅OH and oxygen species resulting from the decomposition of O_3_. The air–PBs + ZnO system resulted in higher TMP degradation (i.e., 95% after 5 min of treatment) compared to the air–PBs + TiO_2_ system (i.e., 87%) and the PBs-alone process (83%). The plasma gas strongly influenced the process, with O_2_ resulting in the best performance and Ar being insufficient to drive the process. The synergy between air–PBs and ZnO was more profound (SF = 1.7), while ZnO also promoted the already high O_2_–plasma bubbles’ performance, resulting in a high TOC removal rate (i.e., 71%). The electrical energy per order in the PBs + ZnO system was very low, ranging from 0.23 to 0.46 kWh/m^3^, depending on the plasma gas and initial TMP concentration. The study provides valuable insights into the rapid and cost-effective degradation of emerging contaminants like TMP and the plasma-catalytic mechanism of antibiotics.

## 1. Introduction

The presence and persistence of antibiotics in aquatic ecosystems have become a major concern in recent years. Antibiotics are widely used as they are essential medications for combating bacterial infections in humans, animals, and plants. However, despite their effectiveness, their abuse, misuse, and improper disposal by various industries, including aquaculture, healthcare, and agriculture, pose major threats to public health and the environment, as their accumulation in water bodies has led to the emergence of antibiotic-resistant bacteria [1,2]. Among these antibiotics, trimethoprim, a widely used medication to treat bacterial infections, has been found to contribute to the contamination of aquatic ecosystems [3,4].

The removal of antibiotics from water is a major challenge due to their persistence and diverse chemical properties. Various water treatment methods have been developed to address this issue [5,6]. However, most of these methods are expensive, are not environmentally friendly, or cannot achieve high removal efficiencies. Advanced oxidation processes (AOPs) are considered more effective in oxidizing a wide range of organic compounds, including antibiotics, as well as being versatile and applicable to various types of wastewater [7]. For instance, photocatalysis has been widely applied for the degradation of antibiotics involving the use of a photocatalyst which becomes activated when exposed to ultraviolet (UV) or visible light [8]. Among the AOPs, non-thermal plasma (NTP) is an emerging and promising process that does not require the use of chemical agents [9,10]. During NTP, a plethora of long- and short-lived reactive species are generated together with UV light and highly energetic electrons that participate in electron transfer reactions with organic molecules, initiating chain reactions that lead to the degradation of pollutants [11,12]. Plasma species include reactive oxygen species (ROS) such as hydroxyl radicals, atomic oxygen, ozone and hydrogen peroxide and reactive nitrogen species (RNS) such as nitrate/nitrite anions, peroxynitrite, etc. [13,14]. Normally, after plasma treatment, the antibiotics are decomposed into less toxic and harmful intermediates, if complete mineralization cannot be achieved [15]. However, the effectiveness of most plasma reactors (e.g., gas–liquid DBD) is limited by the short lifetime of the species and their low penetration into the liquid phase [16]. Therefore, the main challenge remains the uncomplete mineralization of antibiotics resulting from the limited utilization of the UV radiation, electrons and reactive species generated during plasma discharge.

Recently, to alleviate this problem, plasma bubbles have been increasingly explored as a means of enhancing the mass transfer of plasma species from the gas to the aqueous phase [17,18] or in combination with photocatalysts (plasma catalysis) to improve the degradation rate and mineralization of the pollutant by increasing plasma energy utilization [19,20]. During plasma catalysis, the plasma-generated reactive species (electrons, ions, radicals, and UV radiation) can activate the catalyst surface and promote the generation of additional reactive species or convert certain plasma species (e.g., O_3_) to species with higher oxidation potential (e.g., ⋅OH) [21].

In the present study, underwater plasma bubbles, energized by low-frequency high-voltage (HV) nanopulses, were combined with TiO_2_ or ZnO photocatalysts for the degradation of the antibiotic trimethoprim (TMP) in water. Considering that reports on TMP degradation by plasma are scarce [22,23], a thorough investigation was conducted to provide insightful information. Thus, a point-to-point comparison was made between the two catalysts in terms of plasma-catalytic synergy during TMP degradation, species formation and physicochemical properties of the plasma-treated water. In this context, the effect of various parameters in the absence and presence of the two catalysts, such as treatment time, catalyst loading and plasma gas, was investigated. Furthermore, the role of the main plasma species in TMP degradation was evaluated through scavenging experiments, while the process energy efficiency (electrical energy per order) and TMP mineralization were assessed. The strategy of combining underwater plasma bubbles with two well-known photocatalysts could contribute to the understanding of the plasma-catalytic mechanisms that take place during the degradation of antibiotics in water and to the establishment of a highly effective, energy-efficient and rapid process for the destruction of this class of emerging pollutants.

## 2. Materials and Methods

### 2.1. Materials

All reagents were of analytical grade: trimethoprim (TMP, C_14_H_18_N_4_O_3_, M = 290.32 g/mol), ZnO (zinc oxide nanopowder, <100 nm particle size), TiO_2_ (titanium (IV) oxide nanopowder, 21 nm particle size), D-mannitol (D-man), p-benzoquinone (BQ), potassium phosphate monobasic (MP), sodium pyruvate (SP), terephthalic acid (TA), and titanium oxysulfate were purchased from Sigma-Aldrich (St. Louis, MO, USA). Solutions were prepared using Milli-Q water with a resistivity of 18.2 MΩ·cm, obtained by the purification of deionized water, unless otherwise stated. Compressed dry air, oxygen and argon were supplied by Linde (Athens, Greece).

### 2.2. Experimental Setup, Electrical Diagnostics and Treatment Conditions

**Figure 1:**
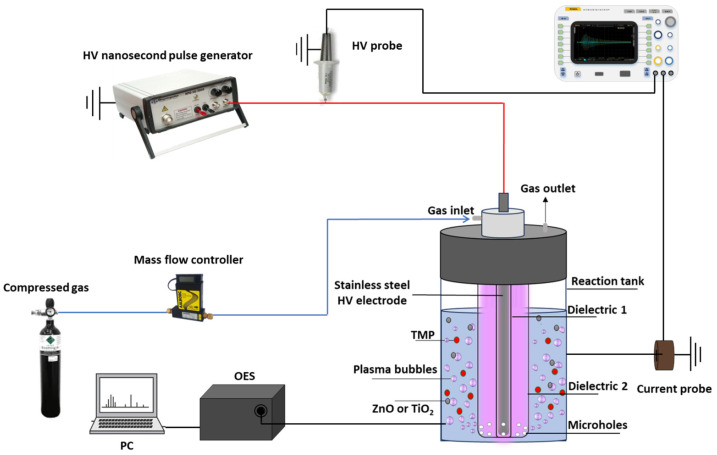
The experimental setup used to treat water samples contaminated by trimethoprim (TMP) and characterize the plasma.

The experimental setup used to treat the TMP-polluted water and characterize the plasma is illustrated in Figure 1. It consisted of (i) a DBD-based plasma bubble reactor driven by (ii) a nanosecond pulsed voltage delivered by a power supply (NPG-18/3500), (iii) a digital oscilloscope (Rigol MSO2302A) connected to voltage (Tektronix P6015A, 0–75 MHz) and current probes (Pearson electronics 2877, 300 Hz–200 MHz) for the discharge power calculation, (iv) an optical characterization arrangement, and (v) a feeding gas system. Details regarding the calculation of the power dissipated in the DBD-based plasma reactor can be found elsewhere [24].

The plasma reactor, based on a coaxial DBD column (Figure 1) capable of delivering plasma bubbles directly into the aqueous solution, was similar to that previously reported [17,18]. Briefly, it consisted of a high voltage stainless-steel rod (5 mm diameter) covered by an inner quartz tube (8.0 mm external diameter) forming a coaxial DBD column with an outer quartz tube (12.0 mm internal diameter). The latter was constructed with 10 microholes uniformly distributed around its base so that the plasma gas, flowing between these dielectric tubes at a constant flow rate (3.0 L/min), could provide plasma bubbles in the solution. The microholes were constructed with a diameter of 400–500 μm to ensure a small bubble size, and therefore a large gas–liquid interfacial area, as a means to increase the mass transfer of short-lived plasma species from the gas to the liquid phase [25]. This coaxial DBD configuration was placed in the center of a quartz reaction tank (46 mm inner diameter, 2 mm wall thickness, 70 mm height), which was filled with the water sample to be treated. A copper tape was attached to the outer surface of the reaction tank and acted as a grounded electrode.

An amount of 70 mL of TMP-polluted water was exposed to plasma treatment (20 sec to 5 min) with an initial pollutant concentration ranging from 5 to 20 mg/L. Pulse voltage and frequency were equal to 25.6 kV and 200 Hz, respectively. For the plasma-catalytic experiments, the catalyst loading ranged from 0 to 0.4 g/L. The solutions containing the catalyst were previously sonicated for 20 min at room temperature. To investigate the effect of plasma chemistry on TMP degradation, different feed gases were introduced into the reactor (air, oxygen and argon). All experiments were carried out in duplicate with excellent reproducibility.

### 2.3. Chemical Analysis of Water Samples

The concentration of TMP was evaluated by high-performance liquid chromatography (HPLC, Shimadzu LC-2050C) equipped with a C18 column (4.6 mm × 50 mm, 3 μm particle size) and a UV detector. According to Liang et al. [23], Aναφορές the temperature was set to 30 °C, detection wavelength to 271 nm and pressure to 100 bar. The flow rate was set to 1.0 mL/min and injection volume to 20 μL. The mobile phase was isocratic and consisted of 0.2 mol/L NaH_2_PO_4_ solution and methanol (70%/30% *v*/*v*). The TMP degradation efficiency, D(%), was calculated as follows:

(1) $$D(\%)=\left[\frac{C_{0}-C_{t}}{C_{0}}\right]\times 100$$

where $C_{0}$ is the initial TMP concentration and $C_{t}$ is its concentration after plasma treatment for a given time.

The degradation rate was described by a pseudo-first-order reaction with respect to the pollutant concentration, and thus, the experimental data were fitted to the following equation:

(2) $$ln\left(\frac{C_{0}}{C_{t}}\right)=k_{app}t$$

where $k_{app}$ (min^−1^) represents the apparent rate constant.

The synergetic factor, namely the synergetic effect between plasma and catalysis, was calculated by the following equation:

(3) $$\mathrm{SF}=\frac{k_{\mathrm{plasma}+\mathrm{catalyst}}}{k_{\mathrm{plasma}}+k_{\mathrm{catalyst}}}$$

where k_plasma+catalyst_ is the rate constant of TMP degradation in the plasma-catalytic system, k_plasma_ is the rate constant in the plasma-alone system, and k_catalyst_ is the rate constant of TMP adsorption onto the catalyst.

The electrical energy per order is the energy required for the decrease in pollutant concentration by one order of magnitude in a unit volume of water and defined as follows:

(4) $$E_{EO}=\frac{Pt}{Vlog\left(\frac{C_{0}}{C_{t}}\right)}$$

where P is the power dissipated in the plasma reactor, V stands for the volume of the treated solution, and t refers to the treatment time.

Finally, a total organic carbon analyzer (Shimadzu, TOC-VCSH) was used to quantify the mineralization of plasma-treated samples. The TOC removal efficiency was calculated by the following equation:

(5) $$TOC(\%)=\left[\frac{{TOC}_{0}-{TOC}_{f}}{{TOC}_{0}}\right]\times 100$$

where ${TOC}_{0}$ and ${TOC}_{f}$ indicate the TOC concentration (mg/L) before treatment and after plasma treatment, respectively.

### 2.4. Identification of Reactive Species in Gas and Aqueous Phase and Use of Scavengers

The critical short- and long-lived reactive oxygen species in the absence and presence of both catalysts were determined. The concentration of hydroxyl radicals (⋅OH) was detected using a photoluminescence spectrometer (Hitachi F2500) Aναφορές [26]. More specifically, the fluorescent properties of 2-hydroxyterephthalic acid (HTA) were monitored, which are produced from the reaction between ⋅OH and terephthalic acid (TA). When the sample was irradiated by UV-A light of 310 nm wavelength, an intensity peak at 425 nm was observed. Furthermore, ozone (O_3_) was quantified by a Hanna multiparameter photometer (HI83399–2), using calibrated reagent kits. Concerning hydrogen peroxide (H_2_O_2_), the titanium oxysulfate method was used [27]. Briefly, 1 mL of titanium oxysulfate solution in 2 M sulfuric acid (25 g/L) was added to 2 mL of each sample, and the absorption peak at 407 nm in the UV-Vis spectrum was quantified (Shimadzu UV-1900). The main atomic and molecular excited plasma species in the gas phase were identified through optical emission spectroscopy (OES) measurements by using a fiber optic spectrometer (AvaSpec-ULS2048CL-EVO, Avantes) in the range of 200–900 nm.

The transient evolution of the physicochemical characteristics of the aqueous solution during plasma treatment was evaluated. The conductivity and pH of the water samples were measured using a Consort multiparameter (Benchtop meter C1020) and a QUANTOFIX^®^ Relax unit (Macherey-Nagel, GmbH), respectively. The initial conductivity of the ultrapure water was equal to 4.6 μScm^−1^ and the pH was 6.5.

To investigate the role of plasma species on the degradation of TMP, scavenging experiments were also conducted. Sodium pyruvate (SP), D-mannitol (D-man), monopotassium phosphate (MP), and p-benzoquinone (BQ) were selected as scavenging agents of H_2_O_2_, ⋅OH, e_aq_^−^ and ⋅O_2_^−^, respectively. The concentration of each scavenger varied from 0.2 to 6 mM to ensure the maximum inhibition of TMP degradation. The action efficiency of plasma species was calculated as follows:

(6) $$\varphi =\left[\frac{D_{0}-D_{1}}{D_{0}}\right]\times 100\%$$

where $D_{1}$ and $D_{0}$ is the TMP degradation in the presence and absence of a scavenger, respectively.

### 2.5. Catalyst Characterization

The crystallographic and pore structure of the catalysts were examined before and after plasma treatment. Plasma-treated catalysts were collected after their exposure to plasma bubbles in the absence of pollutant. The plasma-treated water including the catalyst remained at 80 °C until complete evaporation. The resulting plasma-treated solid (ZnO, TiO_2_) was collected and used for characterization purposes.

The crystal structure of pristine and plasma-treated ZnO and TiO_2_ nanopowder was measured by X-ray powder diffraction (XRD) analysis in the range of 5–90° (2θ). A Bruker D8 Advance diffractometer was used, applying Cu K-alpha radiation (X-ray wavelength λ = 0.15418 nm). The specific surface area and pore size distribution of the pristine and plasma-treated catalysts were measured using a Quantochrome Autosorb IQ-C-MP apparatus. The samples were first degassed at 150 °C for 2 h, and afterwards, nitrogen adsorption and desorption isotherms were obtained at relative pressure P/P0 = 0.05–0.99, where P represents the absolute pressure and P0 the saturation vapor pressure. Using the Brunauer–Emmett–Teller (BET) method, the specific surface area was determined from the nitrogen adsorption (P/P0 in the range 0.05–0.25). Finally, the density functional theory (DFT) method was employed to obtain the volume-based pore size distributions from the whole nitrogen adsorption and desorption isotherms.

## 3. Results

### 3.1. TiO_2_ and ZnO Characterization (XRD, BET) before and after Plasma Treatment and TEM Images

The catalysts were characterized through XRD before and after the plasma bubble treatment. TiO_2_ showed two crystalline phases with a characteristic tetragonal crystal structure, rutile and anatase, as expected. Anatase was the dominant phase (~90%) with apparent peaks at 2theta values of 25.32°, 37.86°, 48.06°, 53.97°, and 55.09°, which are attributed to the (101), (004), (200), (105), and (211) crystal planes, respectively. The rutile phase was observed at minor peaks like 27.45°, 35.98°, and 41.25°, assigned to the (110), (101), and (111) crystal planes, respectively. After plasma treatment, there were no alterations in the structure of TiO_2_ (Figure S1). The XRD pattern of ZnO confirmed its hexagonal wurtzite structure, with the main diffraction peaks at 31.72°, 34.44°, and 36.26° attributed to the (100), (002), and (101) crystal planes, respectively. Similar to TiO_2_, the underwater plasma bubble treatment did not result in changes in the crystal structure of ZnO (Figure S1).

The specific surface area of each catalyst after treatment with nanopulsed plasma bubbles was almost identical to that of the pristine catalysts, with the surface area of TiO_2_ being ~4 times higher compared to that of ZnO (Table 1). Nevertheless, as will be presented below (Section 3.3), the combination of plasma bubbles with ZnO was more effective in degrading TMP than the combination of plasma bubbles with TiO_2_. Furthermore, the plasma-catalytic synergy between the plasma bubbles and catalysts could not be attributed to the modification of the ZnO or TiO_2_ pore structure during treatment, as the plasma bubbles did not induce noticeable changes in the pore volume and pore size distribution of the catalysts (Table 1 and Figure S2).

Figure S3 displays representative TEM images of the TiO_2_ and ZnO samples, providing insight into their respective sizes. The TiO_2_ nanoparticles exhibit a nearly spherical shape, with a mean diameter of approximately 20 nm. Conversely, the ZnO particles display a hexagonal wurtzite structure and possess a mean size of less than 100 nm.

### 3.2. Electrical and Optical Plasma Characterization

**Figure 2:**
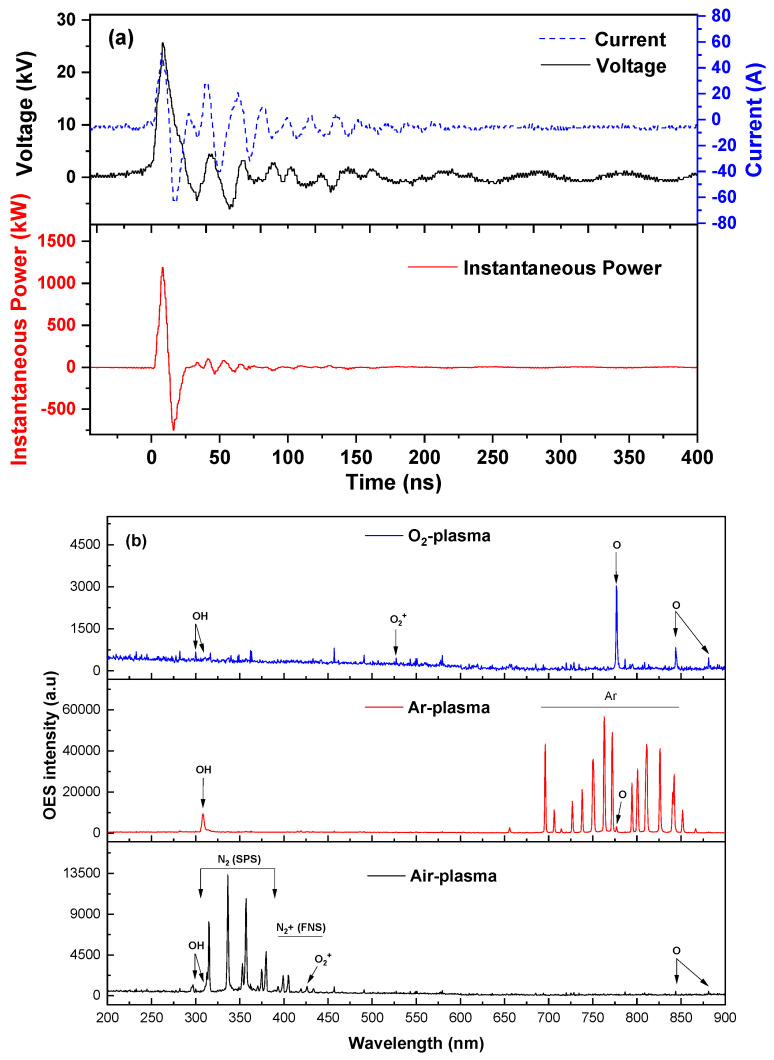
(**a**) Instantaneous voltage, current, and power waveforms during TMP treatment by nanopulsed air–plasma bubbles and (**b**) optical emission spectra of oxygen, argon, and air–plasma in the plasma bubble reactor (pulse voltage: 25.6 kV, pulse frequency: 200 Hz, gas flow rate: 3 L/min).

The electrical power dissipated in the reactor is a measure to evaluate the energy efficiency of the present remediation process. Typical voltage and current nanopulses monitored during TMP treatment by plasma bubbles and the resulting instantaneous power are depicted in Figure 2a. The waveforms initially consist of a high-amplitude pulse followed by smaller-amplitude pulses. The pulse rising time was approximately 4 ns, while the current peak was ~50 A. Despite the applied voltage being a unipolar pulse, the current exhibited bipolar characteristics, with positive pulses (primary discharge) and negative pulses (secondary discharge), attributed to both conduction and displacement current. Although the resulting instantaneous power peak (corresponding to the primary discharge) was very high and equal to ~1.2 MW, the average power dissipated in the plasma bubble reactor was quite low and equal to ~0.8 W due to the very low duty cycle. This is the feature responsible for the better energy efficiency obtained when HV nanosecond pulses are used to energize plasma reactors compared to HV microsecond pulses or sinusoidal HV [18].

Optical emission spectroscopy (OES) was conducted to detect the main excited molecular and atomic plasma species under air, oxygen, and argon atmospheres (Figure 2b). Under O_2_–plasma, the main species identified include the following ROS: an ⋅OH emission peak at 309 nm [28], ⋅O emissions at 777 and 844 nm [15], and weak O_2_^+^ emissions [29]. Under argon–plasma, the spectrum is dominated by multiple argon transition lines (696 to 852 nm), ⋅OH, and weak ⋅O emissions [30]. The OES spectrum obtained during air–plasma treatment is dominated by nitrogen molecules (N_2_—second positive system), nitrogen cation (N_2_^+^—first negative system), ⋅OH, and weak ⋅O and O_2_^+^ emissions [31,32]. The different OES spectra between the various plasma gases result in the generation of different species within the aqueous solution, affecting the degradation of TMP in water (see the results below in Section 3.5).

### 3.3. Effect of Catalyst Addition on Plasma-Treated Water Characteristics

#### 3.3.1. Plasma Species Formation

In dielectric barrier discharge (DBD) plasma, electrons are primarily generated through photoionization and collisional ionization processes in the gas phase. Therefore, an electron colliding with a neutral gas molecule transfers enough energy to ionize the molecule, resulting in the creation of additional electrons and positively charged ions. Subsequently, free radicals capture electrons from neighboring atoms or molecules, leading to the generation of several new chemically active species. The degradation of organic pollutants by plasma lies mainly in the parallel action of reactive oxygen and nitrogen species (RONS). Both short- and long-lived species can be crucial for TMP degradation, and therefore, the concentrations of H_2_O_2_, O_3_, and ⋅OH in the plasma-treated water were measured. The concentration of nitrogen-related species (nitrate, nitrite) was negligible, as previously reported for plasma-treated water inside a DBD-based plasma bubble reactor driven by HV nanopulses [18].

H_2_O_2_ is considered representative among the long-lived plasma species, being mostly formed by recombination reactions between radicals. It is noteworthy that H_2_O_2_ could also lead to the formation of additional oxygen species (R1–R4). A detailed discussion and reactions on the formation of plasma species during water treatment can be found elsewhere [14]:

H_2_O + e^−^ → ⋅H + ⋅OH + e_aq_^−^ (R1)

⋅OH + ⋅OH → H_2_O_2_ (R2)

⋅HO_2_ + ⋅HO_2_ → H_2_O_2_ + O_2_ (R3)

e^−^ + H_2_O_2_ → ⋅OH + OH^−^ (R4)

**Figure 3:**
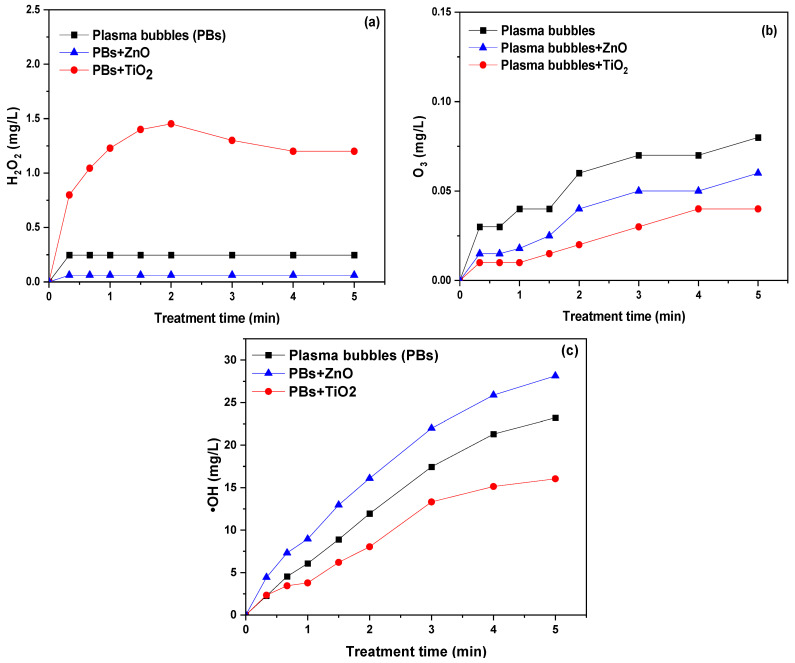
Concentration of plasma species in the absence and presence of TiO_2_ or ZnO. (**a**) H_2_O_2_, (**b**) O_3_, and (**c**) ⋅OH (pulse voltage: 25.6 kV, pulse frequency: 200 Hz, plasma gas: air, flow rate: 3 L/min, catalyst loading: 0.2 g/L).

Compared to the plasma bubbles, the concentration of H_2_O_2_ was decreased in the presence of ZnO and increased substantially in the presence of TiO_2_ (Figure 3a). In particular, after 5 min of treatment with air–plasma bubbles, the H_2_O_2_ concentration was 0.25 mg/L (plasma bubbles), 0.06 mg/L (plasma bubbles + ZnO), and 1.2 mg/L (plasma bubbles + TiO_2_). Therefore, TiO_2_ promoted the reactions that led to the formation of H_2_O_2_, in contrast to ZnO.

Ozone plays a major role in the degradation of pollutants and, therefore, its formation should be quantified. In all cases, O_3_ increased with the treatment time almost linearly, while the presence of both catalysts in the plasma seemed to promote the decomposition of O_3_ (Figure 3b). After 5 min of treatment, the O_3_ concentration decreased from 0.08 mg/L (plasma bubbles) to 0.06 mg/L and 0.04 mg/L in the presence of ZnO and TiO_2_, respectively. The presence of a catalyst may promote the decomposition of O_3_ through various mechanisms: (i) O_3_ is adsorbed on the active sites of ZnO or TiO_2_ and decomposed by the oxygen defects into highly reactive species, like atomic (R5) and singlet oxygen [33,34]; (ii) plasma radiation (*hv*) results in the decomposition of O_3_ into hydrogen peroxide (R6); and (iii) the electrons generated by the irradiation of the photocatalysts by the UV emitted by the plasma can react with O_3_ and reduce it to short-lived and highly reactive **^.^**O_3_^−^, **^.^**O_4_^−^, ^1^O_2_, **^.^**O_2_^−^, and ⋅OH [21].

O_3_ + * → ⋅O + O_2_ (R5)

where * symbolizes the active sites of the catalyst

O_3_ + plasma (*hv*) + H_2_O → H_2_O_2_ + O_2_ (R6)

Among the various plasma species, short-lived ⋅OH is one of the most critical species that interact rapidly with organic pollutants [35,36]. It is directly formed through water dissociation by plasma electrons as well as from the consumption of the long-lived O_3_ and H_2_O_2_:

H_2_O + e^−^ → ⋅H + ⋅OH + e_aq_^−^ (R7)

O_3_ + H_2_O_2_ → ⋅OH + ⋅HO_2_ + O_2_ (R8)

H_2_O_2_ + plasma (*hv*) → 2⋅OH (R9)

Compared to plasma alone, the presence of ZnO enhanced the production of ⋅OH, in contrast to TiO_2_, which had the opposite effect (Figure 3c). For instance, after 5 min of treatment, the concentration of ⋅OH in the aqueous phase treated by plasma bubbles was 23 mg/L and increased to 28 mg/L in the presence of ZnO (plasma bubbles + ZnO), but it decreased to 16 mg/L in the presence of TiO_2_ (plasma bubbles + TiO_2_). The increase in ⋅OH production under ZnO-catalyzed conditions may be attributed to the reaction between the UV-plasma generated holes with water molecules (R10 and R11):

photocatalyst + plasma (*hv*) → e_cb_^−^ + h_vb_^+^ (R10)

h_vb_^+^ + H_2_O → ⋅OH + H^+^ (R11)

On the other hand, the decrease in ⋅OH production under TiO_2_-catalyzed conditions may be attributed to its recombination to form H_2_O_2_ (R2). It is worth reminding here that the concentration of H_2_O_2_ increased in the presence of TiO_2_ (Figure 3a). A possible explanation could be as follows: Given that TiO_2_ has almost quadruple the specific surface area of ZnO and its size is four times smaller than ZnO (Table 1, Figure S3), it is plausible to assume that the active sites in TiO_2_ are more densely packed, making the recombination of ·OH to form H_2_O_2_ (R2) more likely than in ZnO. In other words, in plasma-catalytic wastewater treatment, the use of a photocatalyst with an increased surface area and very small size may potentially lead to the increased production of H_2_O_2_ through the recombination of ·OH. This could explain why H_2_O_2_ increases and ·OH decreases under TiO_2_-catalyzed conditions, while the opposite occurs with ZnO. However, in plasma-catalytic wastewater treatment, the goal is to optimize the conditions that minimize radical recombination to H_2_O_2_, thus maximizing pollutant degradation.

In conclusion, both catalysts promoted the decomposition of the plasma-generated O_3_, probably to other oxygen radicals. The presence of TiO_2_ resulted in a decreased ⋅OH concentration and more H_2_O_2_ compared to plasma alone. In contrast, regarding ZnO, the ⋅OH concentration was enhanced and less H_2_O_2_ was produced compared to plasma alone. It is worth noting that the oxidation potential of H_2_O_2_ is approximately +1.78 volts (V) under standard conditions, whereas the oxidation potential of ·OH is considerably higher, often exceeding +2.8 volts (V) or even higher. These results could contribute to understanding the degradation mechanism of TMP in the presence of either ZnO or TiO_2_. Additionally, identifying the key species involved in degrading specific pollutants can help in selecting the most suitable catalyst for each pollutant.

#### 3.3.2. Physicochemical Water Properties

The aqueous solution’s pH is considered a critical parameter for the effectiveness of plasma in water treatment. Neutral or alkaline pH levels increase the production of ⋅OH due to O_3_ and H_2_O_2_ decomposition, thereby enhancing or reducing pollutant degradation depending on the critical species for its degradation [37,38]. Additionally, alkaline conditions may decrease the oxidation potential of O_3_. Significantly, no substantial fluctuations were observed in the pH during the plasma bubble treatment in the presence or absence of ZnO or TiO_2_ (Figure S4a). This pH stability (~6.5) was further supported by the electrical conductivity of the aqueous solutions (Figure S4b), which showed an insignificant gradual increase with treatment time (from 5 to 20 μS/cm) in all cases.

### 3.4. The Effect of Catalyst Loading on the Degradation of TMP

**Figure 4:**
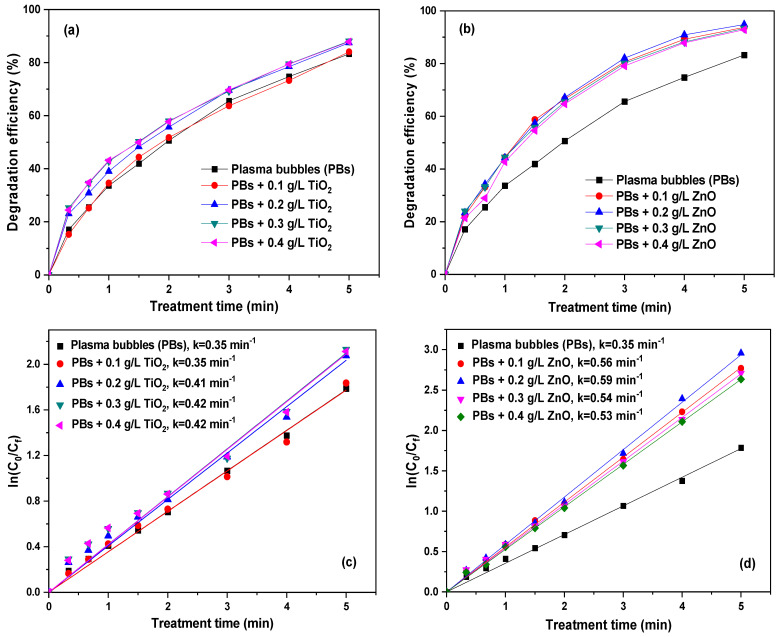
TMP degradation efficiency at various (**a**) TiO_2_ and (**b**) ZnO loadings. Pseudo-first-order degradation kinetics of (**c**) TiO_2_ and (**d**) ZnO (pulse voltage: 25.6 kV, pulse frequency: 200 Hz, plasma gas: air, flow rate: 3 L/min, initial TMP concentration: 20 mg/L).

Numerous studies have investigated the potential synergy between plasma and catalysts for antibiotic degradation [39,40,41,42]. In this study, to harness the UV radiation emitted by plasma discharge, two photocatalysts, ZnO and TiO_2_, were selected. Comparisons of these two catalysts are well documented in other applications and remediation methods [43,44,45,46]. The effect of catalyst loading on TMP degradation was thoroughly investigated by repeating the plasma experiments in the presence of different loadings of ZnO or TiO_2_ (Figure 4). Initially, different loadings of TiO_2_ nanopowder were added (0.1, 0.2, 0.3, and 0.4 g/L). The TiO_2_ catalyst did not appear to significantly enhance the degradation efficiency of TMP (Figure 4a). However, slight changes were observed with varying loadings and when compared to the plasma bubbles-alone system. In particular, there was no notable effect on the degradation of TMP with the addition of 0.1 g/L TiO_2_, which removed approximately ~83.2% of the pollutant in 5 min, similar to the plasma bubbles-alone system. The other three loadings exhibited better degradation efficiency, as depicted in Figure 4a, with the pseudo-first-order degradation kinetics displayed in Figure 4c. Almost 87.4% degradation was achieved, with an apparent rate constant of 0.42 min^−1^. It is worth noting that in terms of TMP removal, 0.2 g/L TiO_2_ was the optimum loading, as higher loadings led to similar TMP degradation. This may be due to agglomerations between TiO_2_ nanoparticles and the potential coverage of its active sites.

Similar experiments were conducted in the presence of ZnO, revealing a different behavior, as ZnO exhibited significant synergy with the plasma bubbles. All ZnO loadings resulted in an enhancement in TMP degradation (Figure 4b). In particular, with the addition of 0.1 and 0.2 g/L ZnO, TMP degradation increased from ~83.2% (plasma bubbles) to ~93.7% and ~94.8%, respectively, after 5 min of treatment. Higher ZnO loadings resulted in a less profound impact, reaching ~93.3% (0.3 g/L) and ~92.8% (0.4 g/L) degradation of TMP. This could be attributed to the agglomeration of the ZnO particles, which are not able to interact properly with the plasma bubbles. The corresponding pseudo-first-order degradation kinetic constants increased from 0.35 min^−1^ (plasma bubbles) to 0.56, 0.59, 0.54, and 0.53 min^−1^, respectively (Figure 4d), indicating that 0.2 g/L is the ideal ZnO loading.

**Figure 5:**
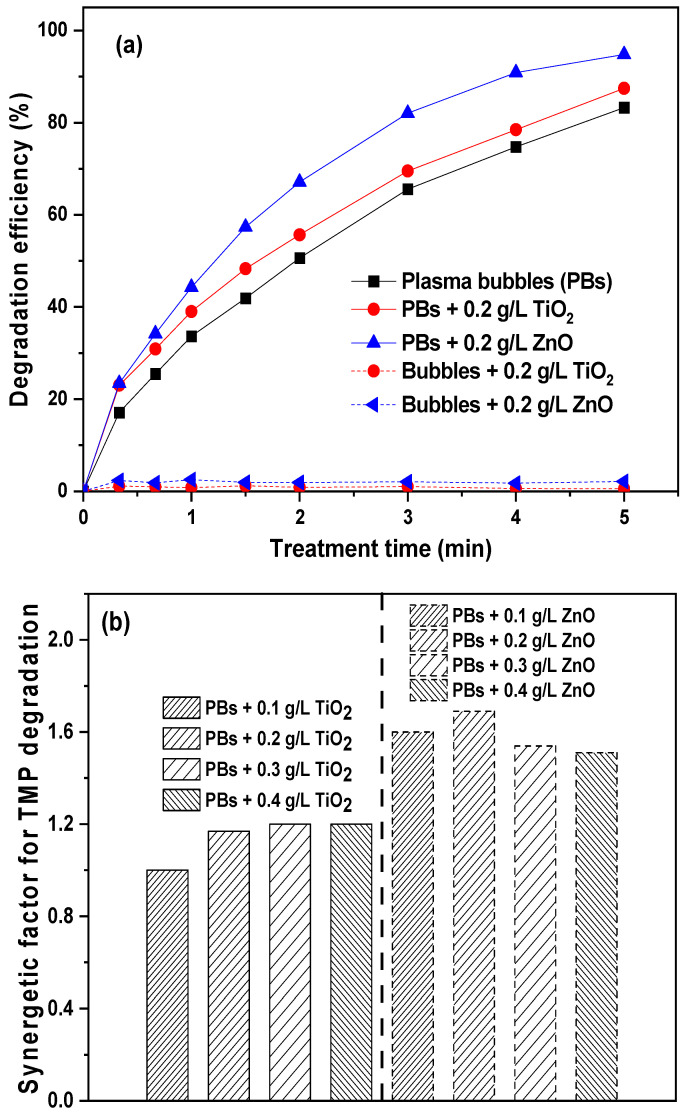
(**a**) Comparison between the TMP degradation efficiencies of plasma bubbles in the presence of TiO_2_ or ZnO (catalyst loading: 0.2 g/L). (**b**) Synergetic factor at various ZnO and TiO_2_ loadings (pulse voltage: 25.6 kV, pulse frequency: 200 Hz, plasma gas: air, flow rate: 3 L/min, initial TMP concentration: 20 mg/L).

A direct comparison between the two catalysts under their optimal loading is presented in Figure 5a. It is evident that TMP degradation under plasma bubbles + ZnO was higher compared to the plasma bubbles + TiO_2_ system and plasma bubbles-alone process. For instance, TMP degradation at the end of treatment (i.e., 5 min) was ~83.2% (plasma bubbles), ~94.8% (plasma bubbles + ZnO), and ~87.4% (plasma bubbles + TiO_2_). Additionally, negligible TMP adsorption onto both catalysts was observed under no plasma conditions (only bubbles with a catalyst).

Utilizing the previously calculated apparent rate constant, the synergistic factor (SF) was deduced for each catalyst at every loading, as depicted in Figure 5b. The corresponding SF in the presence of ZnO was 1.56, 1.70, 1.54, and 1.53 for 0.1, 0.2, 0.3, and 0.4 g/L, respectively, while the corresponding values for TiO_2_ were 1, 1.14, 1.17, and 1.17. Therefore, the highest SF achieved was 1.7 with 0.2 g/L ZnO, which is approximately 1.5 times higher than TiO_2_ at the same loading. Consequently, ZnO appears to be much more beneficial when combined with plasma bubbles for the degradation of TMP. Interestingly, ZnO exhibited increased plasma-catalytic performance compared to TiO_2_ despite its lower surface area (Table 1). The superiority of ZnO can be rationalized by the vital role of plasma species in the TMP degradation process (see discussion below in Section 3.7) and their relative concentration, which in turn depends on the type of the catalyst (as discussed in Section 3.3.1).

### 3.5. The Effect of Plasma Gas and Initial TMP Concentration on Its Degradation by Plasma Bubbles in the Absence and Presence of ZnO

**Figure 6:**
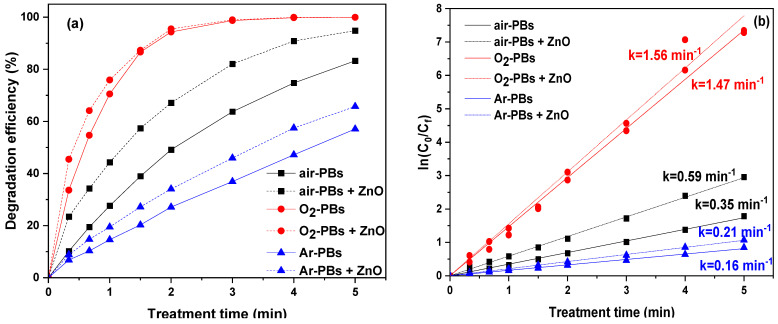
(**a**) TMP degradation as a function of treatment time under air–plasma bubbles, O_2_–plasma bubbles, and Ar–plasma bubbles in the presence and absence of ZnO and (**b**) corresponding pseudo-first-order degradation kinetics (pulse voltage: 25.6 kV, pulse frequency: 200 Hz, flow rate: 3 L/min, initial TMP concentration: 20 mg/L, ZnO loading: 0.2 g/L).

The effectiveness of plasma for water treatment depends on numerous parameters related to plasma conditions and chemistry [10]. Plasma chemistry is related to the nature of the produced reactive oxygen and nitrogen species (RONS), which depend on the gas used for the discharge formation [47]. Altering the plasma gas can result in the production of different types and concentrations of RONS, which play a major role in pollutant degradation. For this reason, the effect of plasma gas (air, oxygen, and argon) on TMP degradation was investigated under the conditions of plasma bubbles in the absence and presence of ZnO, which proved to be a more effective catalyst for TMP degradation (Figure 6). When oxygen and argon were used instead of air as the feeding gas, a different TMP degradation profile was observed. Under O_2_–plasma bubbles, very high TMP degradation (i.e., ~94%) was recorded after only 2 min of treatment, whereas the addition of ZnO further promoted this performance to ~96% (Figure 6a). This higher performance of oxygen is related to the enhanced reactive oxygen species (ROS) production under an O_2_-containing atmosphere, indicating the significance of ROS over reactive nitrogen species (RNS) in the TMP degradation process.

On the other hand, under argon–plasma bubbles, a lower TMP degradation was achieved (~57%) even after 5 min of treatment, while the addition of ZnO increased the TMP degradation to 65% (Figure 6a). It could, therefore, be assumed that the reactions and species promoted by argon, such as electrons, argon ions, and ⋅OH, are also involved in TMP degradation but not as the major species. It is also interesting to note that the impact of ZnO with the different gases was not the same. The apparent rate constant of air–plasma bubbles + ZnO was ~1.6 times higher compared to that of air–plasma bubbles, being 0.59 and 0.35 min^−1^, respectively (Figure 6b). Concerning argon, the apparent rate constant increased from 0.16 to 0.21 min^−1^ (~1.3 times higher). In addition, considering the already very high performance of O_2_–plasma bubbles, the presence of ZnO did not result in a significant enhancement in the degradation rate of the parent TMP molecule (1.47 to 1.56 min^−1^). Nevertheless, the TOC measurements presented below (Section 3.8) are indicative of the plasma-catalytic synergy between ZnO and O_2_–plasma bubbles. Based on the apparent rate constants, the synergistic factor (SF) values for air–plasma bubbles + ZnO and argon–plasma bubbles + ZnO were 1.70 and 1.31, respectively. A possible explanation for why the effect of ZnO was more intense in the case of air could lie in the fact that the absorption spectrum of ZnO (UVA/UVB region) overlaps with the corresponding radiation emitted by air–plasma. On the other hand, the radiation emitted from argon is mainly in the VUV/visible light region, meaning the photoactivation of the catalyst is less effective.

**Figure 7:**
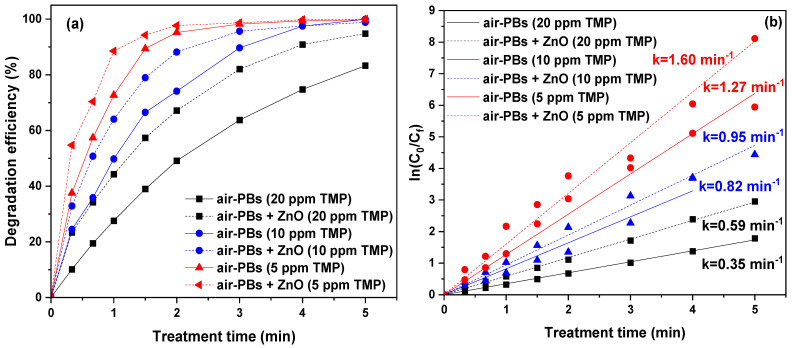
(**a**) TMP degradation as a function of treatment time under air–plasma bubbles at various initial TMP concentrations in water in the presence and absence of ZnO and (**b**) corresponding pseudo-first-order degradation kinetics (pulse voltage: 25.6 kV, pulse frequency: 200 Hz, flow rate: 3 L/min, ZnO loading: 0.2 g/L).

The effect of the initial pollutant concentration on its degradation was extensively investigated. Nonetheless, from a practical standpoint, with the inclusion of this analysis in the present study, we aimed to assess the efficacy of the current approach across a range of pollutant concentrations in water, encompassing both higher and lower (and more realistic) levels of TMP contamination in water. Therefore, the air–plasma bubbles process was tested under various initial TMP concentrations, both with and without ZnO. For this purpose, experiments were conducted using initial TMP concentrations of 20 mg/L, 10 mg/L, and 5 mg/L, with the results depicted in Figure 7. Evidently, the lower the initial TMP concentration, the higher its degradation efficiency. For 20 mg/L TMP, 83.2% degradation was achieved after 5 min of treatment, while complete degradation (99.9%) was achieved at an initial concentration of 10 mg/L. For the lowest concentration (5 mg/L), complete degradation was also achieved but with a lower treatment time (4 min). The addition of ZnO apparently enhanced the TMP degradation, as reflected in the apparent degradation rate constants (Figure 7b). In particular, for 20 mg/L TMP, the rate increased by ~1.7 times from 0.35 min^−1^ to 0.59 min^−1^. At a lower concentration, 10 mg/L, it increased from 0.82 min^−1^ to 0.95 min^−1^, whereas for an even lower concentration of TMP, 5 mg/L, the constant was ~1.3 times higher with the addition of ZnO.

### 3.6. Process Energy Efficiency

**Figure 8:**
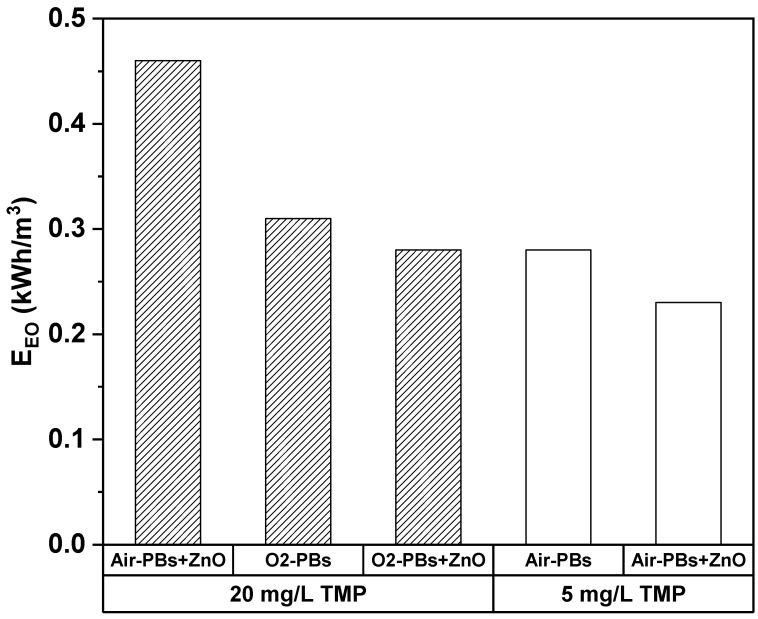
Electrical energy per order (E_EO_) for the TMP degradation in water under air– and oxygen–plasma bubbles in the absence/presence of ZnO (pulse voltage: 25.6 kV, pulse frequency: 200 Hz, flow rate: 3 L/min, initial TMP concentration: 5 or 20 mg/L, ZnO loading: 0.2 g/L).

The energy efficiency of a process is a critical factor in determining its broader applicability as a treatment method. One measure used to evaluate process effectiveness is electrical energy per order (E_EO_), which considers the discharge power, treated solution volume, and treatment time needed to reduce the pollutant concentration by at least one order of magnitude. In this study, the E_EO_ for TMP degradation at two different initial concentrations in water (5 and 20 mg/L) under air– and oxygen–plasma bubbles, in the absence and presence of ZnO, is depicted in Figure 8.

For 20 mg/L TMP, the E_EO_ under air– and O_2_–plasma bubbles combined with ZnO was 0.46 and 0.28 kWh/m^3^, respectively. The air–plasma bubbles-alone system failed to achieve degradation higher than 90%, while the E_EO_ under O_2_–plasma bubbles was also very low (i.e., 0.31 kWh/m^3^). Even lower energy requirements were observed for an initial TMP concentration of 5 mg/L under air–plasma bubbles combined with ZnO (0.23 kWh/m^3^), which is significant considering its closer resemblance to real-life conditions. The E_EO_ of this study is compared to other AOPs (for TMP degradation) and to other plasma-catalytic studies (for the treatment of water polluted by antibiotics) in Table 2, as this study is the first attempt to combine plasma with catalysis for TMP degradation. Specifically, Chen et al. investigated the degradation of amoxicillin by DBD plasma combined with a Ce_0.5_Bi_0.5_VO_4_ catalyst on a honeycomb ceramic plate, reporting an increase in amoxicillin degradation from 30% (DBD alone) to 65%, with the corresponding E_EO_ being 79.4 kWh/m^3^ [48]. Xiao et al. combined DBD with a ZnO/cellulose acetate film catalyst for the degradation of sulfadiazine, reporting an E_EO_ of 15.4 kWh/m^3^ [49]. Regarding TMP degradation, the E_EO_ achieved by the combination of plasma bubbles with ZnO is significantly lower compared to other AOPs (Table 2), highlighting the potential of this combination for the rapid and effective degradation of antibiotics in water.

### 3.7. The Role of Plasma Species in the Degradation of TMP by Plasma Bubbles

To understand the mechanism of TMP degradation by plasma bubbles, it was essential to determine the contribution of the main plasma species to its degradation. Therefore, the role of ⋅OH, ⋅O_2_^−^, e_aq_^−^, and H_2_O_2_ was explored by using D-mannitol (D-man), p-benzoquinone (BQ), monopotassium phosphate (MP), and sodium pyruvate (SP), respectively, as suitable scavengers. The selected optimum concentration of each scavenger was the minimum concentration at which no further inhibition of TMP degradation was observed.

**Figure 9:**
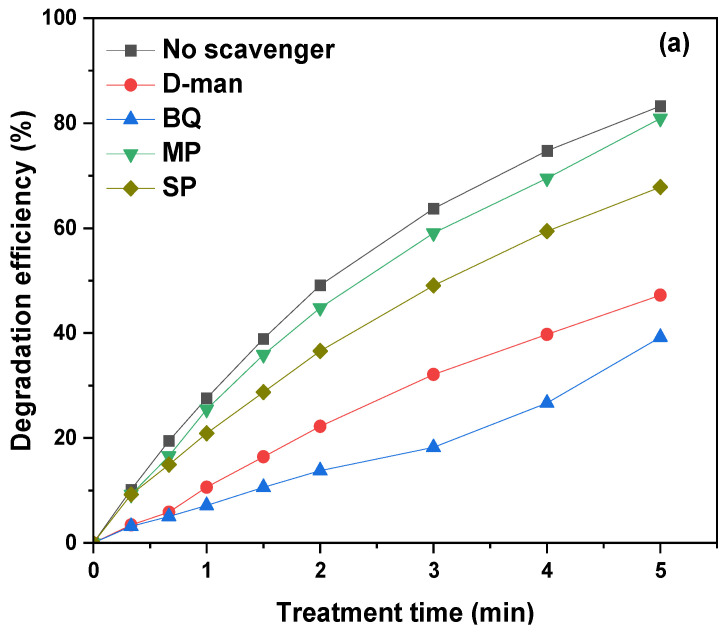

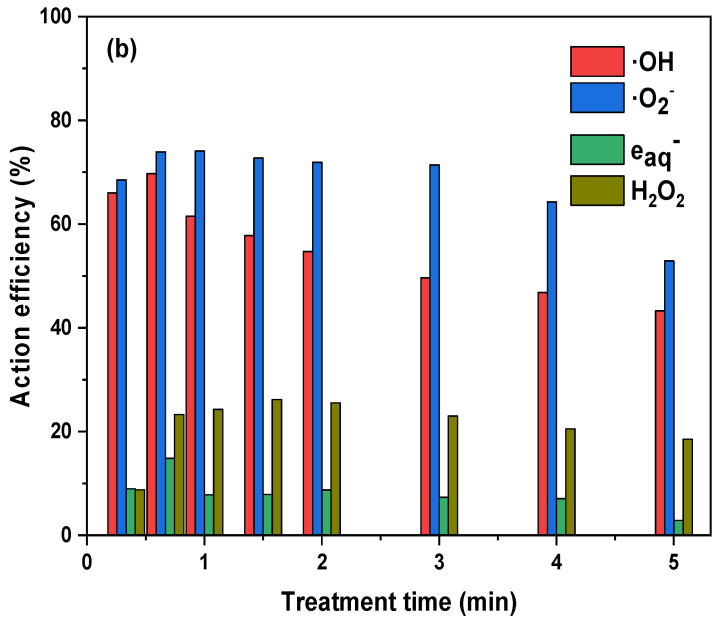
(**a**) Effect of scavengers on TMP degradation by plasma bubbles and (**b**) action efficiency of plasma species (pulse voltage: 25.6 kV, pulse frequency: 200 Hz, plasma gas: air, flow rate: 3 L/min, initial TMP concentration: 20 mg/L, D-man concentration: 6 mM, BQ concentration: 1 mM, MP concentration: 1 mM, SP concentration: 1 mM).

It is evident that the presence of all scavengers inhibited the degradation of TMP, indicating that all these species contributed to its degradation (Figure 9a). For instance, after 4 min of plasma bubble treatment, the degradation of TMP decreased from 74.7% (no scavenger) to 69.5%, 59.4%, 39.7%, and 26.7% in the presence of MP, SP, D-man, and BQ, respectively. Therefore, hydrated electrons and H_2_O_2_ contributed the least to TMP degradation, while ⋅OH proved to play a considerable role in the process, with the most significant contribution coming from ⋅O_2_^−^. Depending on the treatment time, the calculated action efficiency of ⋅O_2_^−^ ranged between ~53 and 74%, while the corresponding action efficiencies of ⋅OH, H_2_O_2_, and e_aq_^−^ ranged between ~43 and 70%, ~9 and 26%, and ~3 and 15%, respectively (Figure 9b). The significantly more pronounced role of ·OH in the degradation of TMP compared to H_2_O_2_ justifies the superior plasma-catalytic performance of ZnO compared TiO_2_. It is worth reminding here that in the presence of TiO_2_, the concentration of H_2_O_2_ was increased, while the concentration of ·OH was decreased compared to plasma alone. Conversely, with ZnO, the presence of this substance led to a decrease in H_2_O_2_ concentration and an increase in ·OH concentration (Figure 3a,c).

### 3.8. The Mineralization of TMP under Plasma Bubbles in the Absence and Presence of ZnO

**Figure 10:**
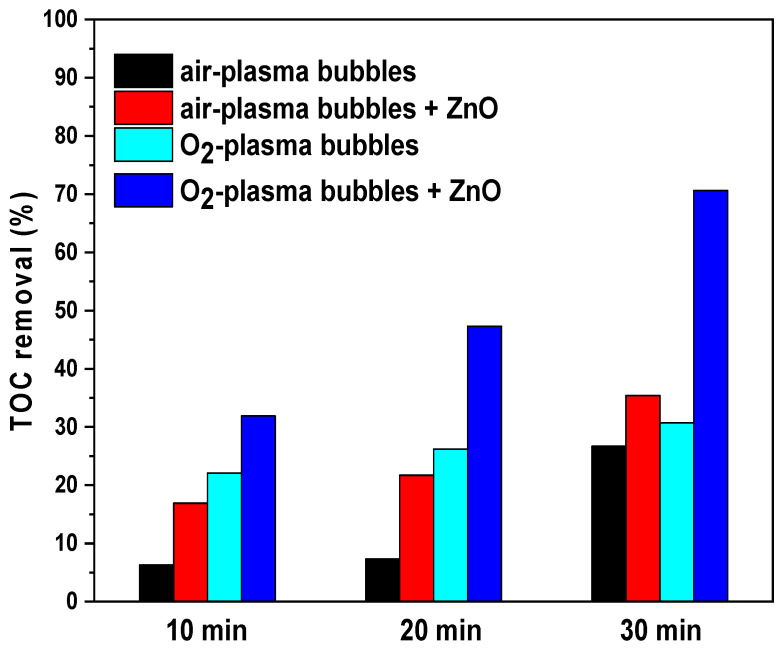
TOC removal during TMP degradation by air– and O_2_–plasma bubbles in the presence/absence of ZnO (pulse voltage: 25.6 kV, pulse frequency: 200 Hz, flow rate: 3 L/min, initial TMP concentration: 20 mg/L, ZnO loading: 0.2 g/L).

The effectiveness of this remediation approach for TMP degradation was explored in terms of total organic carbon (TOC) removal. The samples tested were from the following systems: air–plasma bubbles, air–plasma bubbles + ZnO, O_2_–plasma bubbles, and O_2_–plasma bubbles + ZnO for 10, 20, and 30 min of plasma treatment. ZnO was beneficial to TOC removal for both the air–plasma bubbles and O_2_–plasma bubbles treatments (Figure 10). In particular, the TOC removal rate for air–plasma bubbles was 27% after 30 min, while in the presence of ZnO, it increased significantly to 35% with a shorter treatment time (i.e., 20 min). In the case of the O_2_–plasma bubbles treatment, the TOC removal rate remained stable at ~30% regardless of the treatment time, while the addition of ZnO resulted in a gradual increase with treatment time, reaching an impressive TOC removal rate of 71% after 30 min of treatment. Therefore, ZnO-catalyzed plasma bubbles resulted in the complete removal of the TMP parent molecule but also in noticeable TOC removal.

## 4. Conclusions

In this study, a novel plasma-catalytic process was proposed for degrading trimethoprim (TMP) in water by combining underwater plasma bubbles with ZnO or TiO_2_ photocatalysts. The plasma bubbles were energized by high-voltage nanosecond pulses to enhance the production yield of plasma species. Both catalysts maintained their pore structure after plasma bubble treatment, and the physicochemical properties of water (pH and electrical conductivity) remained unaffected.

ZnO and TiO_2_ both facilitated the decomposition of plasma-generated O_3_ in water to other oxygen radicals. However, TiO_2_ led to a decrease in ⋅OH concentration and an in-crease in H_2_O_2_, while ZnO resulted in an increased ⋅OH concentration and a decrease in H_2_O_2_. ZnO-catalyzed plasma bubbles outperformed TiO_2_-catalyzed ones in TMP degradation, achieving 94.8% degradation compared to 87.4% with TiO_2_ after 5 min of treatment. The synergetic factor was 1.70 for ZnO and 1.17 for TiO_2_, indicating that the synergy was ~1.5 times higher for ZnO.

TMP degradation was higher under oxygen– and air–plasma bubbles compared to argon–plasma bubbles, with ZnO-catalyzed air–plasma bubbles showing effectiveness across various initial TMP concentrations. The plasma-catalytic synergy was higher in air–plasma bubbles compared to argon–plasma bubbles (with synergistic factors of 1.70 and 1.31, respectively), which aligns with the wavelength of UV light produced by each plasma gas and the UV absorption range of ZnO. ⋅O_2_^−^ and ⋅OH were the primary contributors to TMP degradation, with ⋅OH playing a more significant role than H_2_O_2_. This justified the superior plasma-catalytic performance of ZnO compared to TiO_2_.

The combination of HV nanopulses with plasma bubbles and ZnO proved to be optimal for minimizing energy costs, with the calculated electrical energy per order (E_EO_) ranging from 0.23 to 0.46 kWh/m^3^. The ZnO-catalyzed O_2_–plasma bubbles achieved a remarkable 71% removal rate of TOC after 30 min of treatment. These findings underscore the potential of this study in contributing to the development of highly effective processes for antibiotic degradation in water.

## Figures and Tables

**Table 1:** Specific surface area and pore volume of TiO_2_ and ZnO before and after plasma bubble treatment (pulse voltage: 25.6 kV, pulse frequency: 200 Hz, plasma gas: air, gas flow rate: 3 L/min, treatment time: 5 min, catalyst loading: 0.2 g/L).

Specific Surface Area (m^2^/g)/Pore Volume (cm^3^/g)		
Catalyst	Pristine	Plasma-Treated
TiO_2_	54.96/0.121	54.92/0.160
ZnO	13.18/0.024	13.01/0.026

**Table 2:** Comparison of this study with other AOPs for the degradation of antibiotics in water.

Plasma Process	Pollutant	Degradation Efficiency (%)	Treatment Time (min)	E_EO_ (kWh/m^3^)	Ref.
DBD plasma + Ce_0.5_Bi_0.5_VO_4_	Amoxicillin 50 mg/L	94.5	30	79.4	[47]
DBD plasma + ZnO on cellulose acetate films	Sulfadiazine 20 mg/L	97.2	60	15.4	[48]
Plasma + graphene–TiO_2_–Fe_3_O_4_	Oxytetracycline 40 mg/L	98.1	60	-	[49]
DBD + Cu-CeO_2_@CA	Ciprofloxacin 200 mg/L	89.5	40	-	[50]
UV–chloride (1 mM)	Trimethoprim 2.9 mg/L	91	20	3.6	[51]
Photocatalysis (TiO_2_ film irradiated with simulated solar radiation)	Trimethoprim 10 mg/L	90	101.5	8441.4	[52]
Nanopulsed air–plasma bubbles + ZnO	Trimethoprim 20 mg/L	90.9	4	0.46	This study
Nanopulsed O_2_–plasma bubbles	Trimethoprim 20 mg/L	94.3	2	0.31	This study
Nanopulsed O_2_–plasma bubbles + ZnO	Trimethoprim 20 mg/L	95.5	2	0.28	This study
Nanopulsed air–plasma bubbles + ZnO	Trimethoprim 5 mg/L	94.2	1.5	0.23	This study

## Data Availability

No new data were created or analyzed in this study. Data sharing is not applicable to this article.

## Supplementary Materials

The following supporting information can be downloaded at: https://www.mdpi.com/article/10.3390/nano14100815/s1, Figure S1. The XRD patterns of (a) TiO_2_ and (b) ZnO before and after plasma bubble treatment (pulse voltage: 25.6 kV, pulse frequency: 200 Hz, plasma gas: air, flow rate: 3 L/min, treatment time: 5 min, catalyst loading: 0.2 g/L). Figure S2. Pore size distribution of (a) ZnO and (b) TiO_2_ before and after plasma bubble treatment (pulse voltage: 25.6 kV, pulse frequency: 200 Hz, plasma gas: air, flow rate: 3 L/min, treatment time: 5 min, catalyst loading: 0.2 g/L). Figure S3. TEM images of (a) TiO_2_ nanoparticles and (b) ZnO nanopowder. Figure S4. (a) pH and (b) conductivity of plasma-treated water as a function of plasma bubble treatment time in the absence or the presence of catalysts (pulse voltage: 25.6 kV, pulse frequency: 200 Hz, plasma gas: air, flow rate: 3 L/min, catalyst loading: 0.2 g/L).
