# Supplementary material for: Exploring the Synergistic Mechanisms of Nanopulsed Plasma Bubbles and Photocatalysts for Trimethoprim Degradation and Mineralization in Water
Source: Nanomaterials (Basel). 2024 May 7;14(10):815. doi: 10.3390/nano14100815 (PMC11123754; doi:10.3390/nano14100815)
Supplement: Supplementary file 1 [file nanomaterials-14-00815-s001.zip › nanomaterials-2968828-supplementary.pdf]

Exploring the synergistic mechanisms of nanopulsed plasma bubbles and photocatalysts for trimethoprim degradation and mineralization in water

Dimitris Tsokanas[1,2] and Christos A. Aggelopoulos[1,*]

1 Laboratory of Cold Plasma and Advanced Techniques for Improving Environmental Systems, Institute of Chemical Engineering Sciences, Foundation for Research and Technology Hellas (FORTH/ICE-HT), 26504, Patras, Greece
2 Chemistry Department, University of Patras, 26504, Patras, Greece
* Correspondence: caggelop@iceht.forth.gr; Tel.: +30 2610965205

S1 XRD characterization of the catalyst

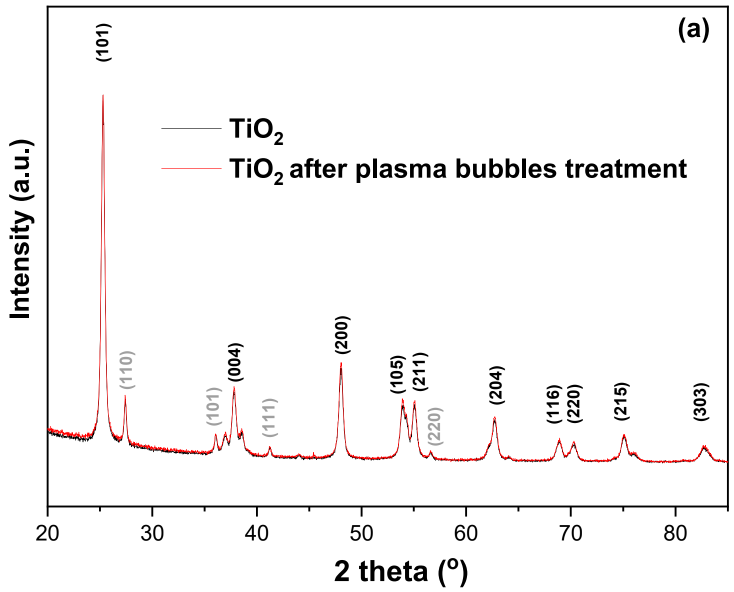

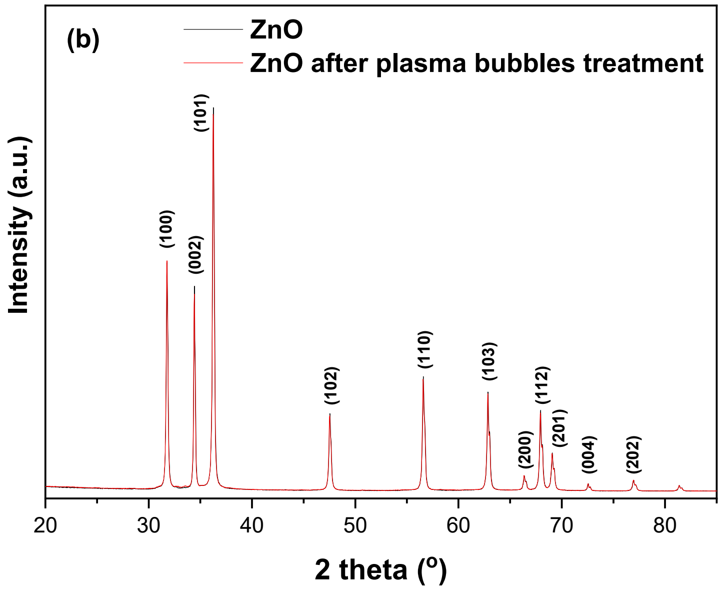

Figure S1. The XRD patterns of (a) TiO_2 and (b) ZnO before and after plasma bubbles treatment (pulse voltage: 25.6 kV, pulse frequency: 200 Hz, plasma gas: air, flow rate: 3 L/min, treatment time: 5 min, catalyst loading: 0.2 g/L).

S2 BET analysis

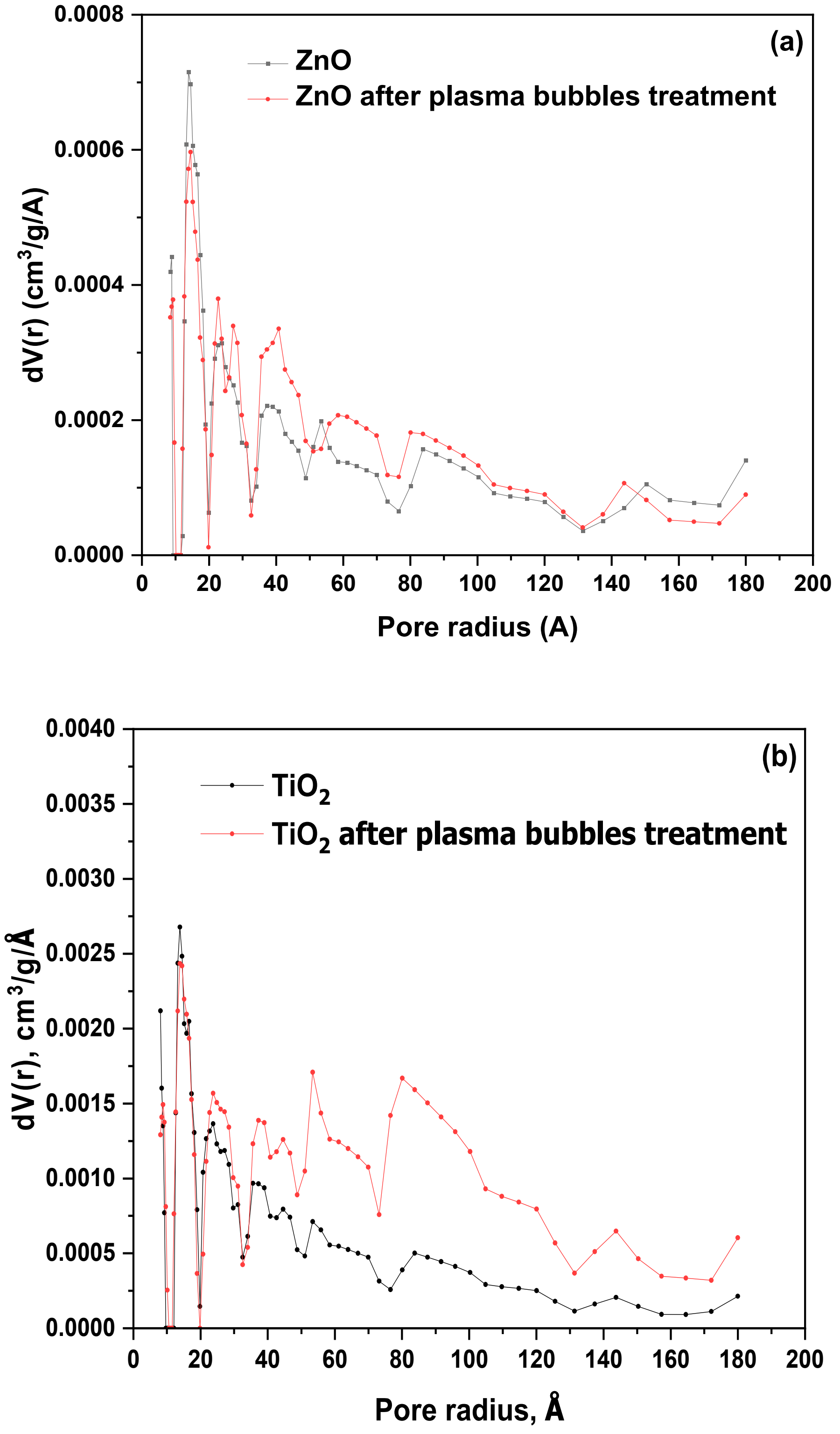

Figure S2. Pore size distribution of (a) ZnO and (b) TiO_2 before and after plasma bubbles treatment (pulse voltage: 25.6 kV, pulse frequency: 200 Hz, plasma gas: air, flow rate: 3 L/min, treatment time: 5 min, catalyst loading: 0.2 g/L).

S3 TEM images

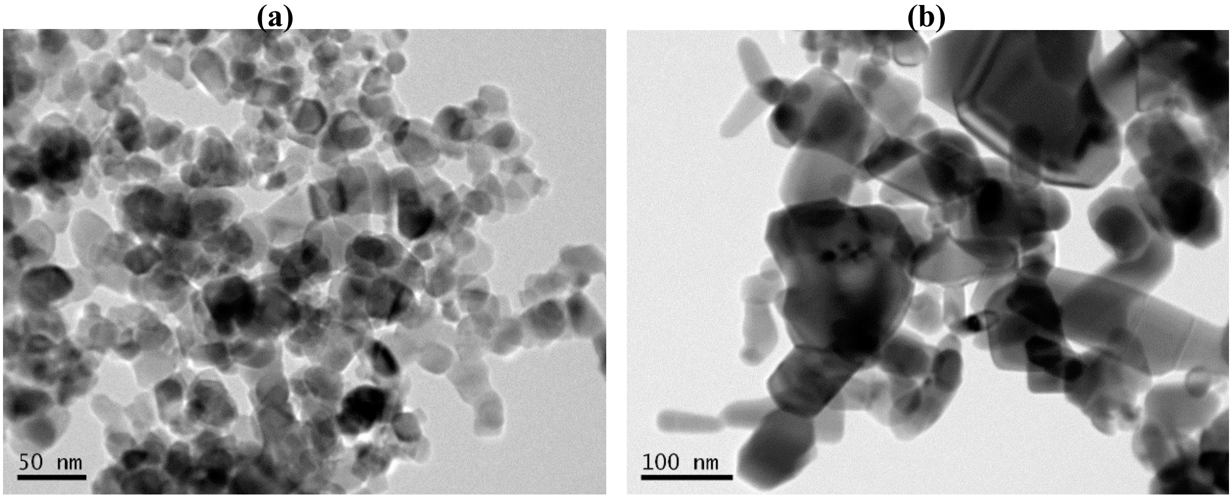

Figure S3. TEM images of (a) TiO2 nanoparticles and (b) ZnO nanopowder.

S4 pH and conductivity measurements

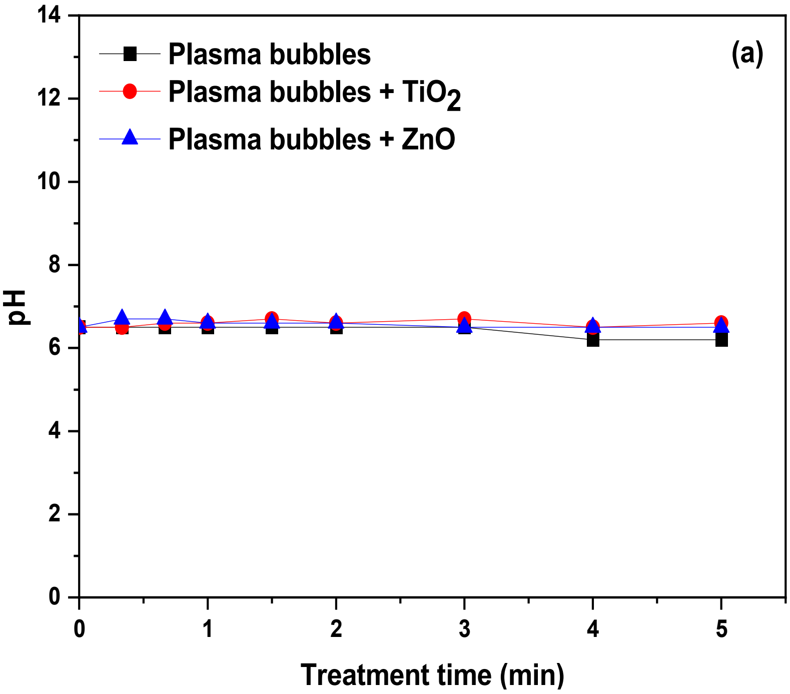

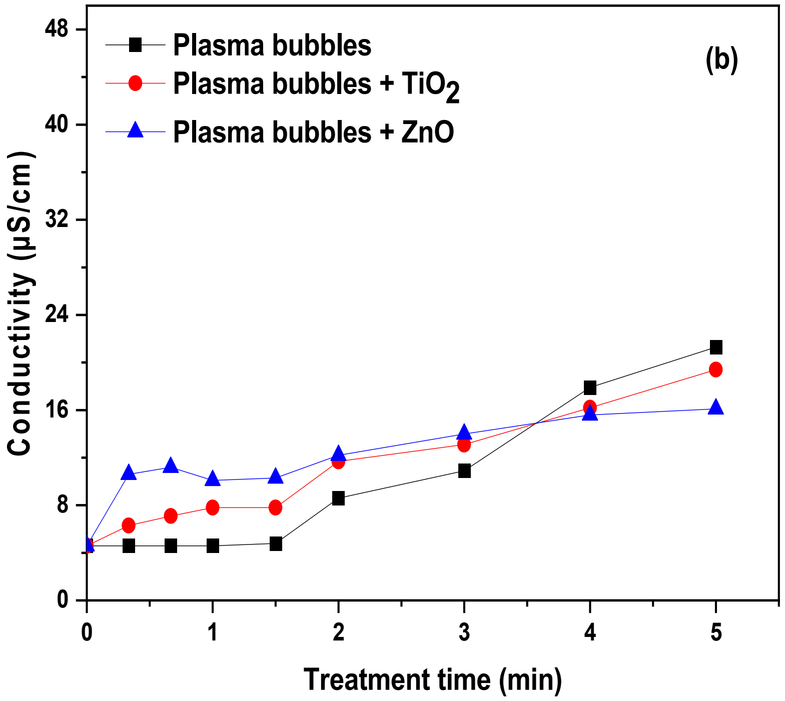

Figure S4. (a) pH and (b) conductivity of plasma treated water as a function of plasma bubbles treatment time in the absence or the presence of catalysts (pulse voltage: 25.6 kV, pulse frequency: 200 Hz, plasma gas: air, flow rate: 3 L/min, catalyst loading: 0.2 g/L).
